# Why Hasn’t the Gifted Label Caught up with Science?

**DOI:** 10.3390/jintelligence10040084

**Published:** 2022-10-12

**Authors:** Michael S. Matthews, Jennifer L. Jolly

**Affiliations:** 1 Department of Special Education & Child Development, Cato College of Education, University of North Carolina at Charlotte, 9201 University City Blvd., Charlotte, NC 28223, USA; 2 Autherine Lucy Hall, University of Alabama, Tuscaloosa, AL 35487, USA; 3 Morven Brown Hall, University of New South Wales, Sydney, NSW 2031, Australia

**Keywords:** gifted education, history of education, nature vs. nurture

## Abstract

The development of both special education and gifted education as fields of study were closely tied to the origins of intelligence testing in the early 20th century. While special education’s terminology has become more nuanced and circumspect over the ensuing century, the term *gifted* has remained unchanged despite coming under substantial criticism in recent decades for its lack of specificity and for the innateness that the term implies as the primary cause of individual differences in ability. We examine this history and the seminal nationally disseminated reports related to gifted education, from the Marland report to the present, to consider why the gifted label has persisted. We conclude with some suggestions for how these issues might be remedied.

## 1. Introduction

By the close of the 1920s, both special education and gifted education had emerged as subfields of study within psychology and educational psychology. Now approximately one hundred years later, this anniversary offers an important opportunity to reflect on how our knowledge has grown and changed over the ensuing century. How have the changes in our knowledge of intelligence and its measurement over the past 100 years informed special and gifted education practice? How and why has the terminology of special education changed so thoroughly since its origins, while the often-problematic label ‘gifted’ has stubbornly persisted since its origin prior to Lewis Terman’s seminal 1925 publication (Terman 1925)? We attempt to address the first question by summarizing major developments in both our understanding of intelligence and in gifted education over the past century. This is followed by a synthesis of their relevance for understanding the current state of gifted education and where the field might be headed.

## 2. Background

### 2.1. Development of Ideas about Intelligence

Attempts to quantify the measurement of intelligence have a lengthy history that we are only able to touch upon briefly. One general trend is that over time, ideas about what intelligence is have become both more nuanced and better understood. Spearman, working in the first three decades of the 20th century, felt that there was a single factor (named “g” for general intelligence) that could explain the commonality of performance across different measures of intelligence (Spearman 1904); a factor of specific intellectual abilities (“s”) explained the remainder. Thorndike (1926) suggested that “for ordinary practical purposes, however, it suffices to examine for three ‘intelligences,’ which we may call mechanical intelligence, social intelligence, and abstract intelligence” (p. 228). Spearman later divided g into eductive and reproductive abilities, both of which he considered to be primarily genetic in origin. As an aside, Spearman also observed late in his career that the degree of correlation across different measures decreased as overall IQ score increased; this observation is still being studied (e.g., Kane and Oakland 2000), but may also be relevant to understanding the development of gifted education practice.

Efforts to measure intelligence developed in tandem with the development of more sophisticated statistical methods in psychology. Using factor analysis, Thurstone (1938) suggested the existence of seven primary mental abilities that he believed provided a better fit to his data than Spearman’s g did. Of relevance to gifted identification practice, Thurstone also developed the standardized mean and standard deviation score reporting for IQ; this method replaced Binet’s original age-ratio interpretation, and it is still used today to report IQ scores. When writers in gifted education have described the use of an IQ score cutoff to identify giftedness (e.g., McBee and Makel 2019), it is this standardized mean scale and full-scale IQ score that typically are being referenced even when this is not fully explained.

Guilford also rejected Spearman’s conceptualization of a general factor of intelligence; in his presidential address to the APA, Guilford proposed 120 different aspects of intelligence (later expanded to 150) in his Structure of the Intellect model (Guilford 1956). Of specific relevance to gifted education, this work was among the first to propose a relationship between intelligence and creativity, influencing among others the career and research of E. Paul Torrance. Since that time, creativity consistently has been included in most major theoretical definitions of giftedness yet has been conspicuously absent from gifted identification practice in most settings (Matthews 2015).

More recent conceptualizations of intelligence typically have far fewer different categories than Guilford’s model did, though Gardner’s multiple intelligences model (which has not found empirical support) suggested more than ten discrete areas of intelligence. However, individually administered intelligence tests have not necessarily followed this trend toward simplification. Though a composite or full-scale IQ score is still provided, the WISC-V (for example) has evolved from having 12 subtests in its first version in 1949, to now being composed of 21 subtests (Wechsler 2014); however, the two-component Cattell-Horn-Carroll theory (Gf-Gc; fluid and crystalized intelligence) as an explanation of cognitive abilities has gained prominence in informing the theoretical underpinnings of many contemporary IQ tests (McGill and Dombrowski 2019).

As the conception of intelligence has expanded over the past century, the term gifted has remained static despite a growing recognition of the need for additional data beyond the IQ score to inform the gifted identification process (Baldwin 1977; McBee and Makel 2019). Additionally, the term gifted, on its own, does not provide any educational guidance.

Guy M. Whipple is credited with being the first to use the term gifted (Henry 1920). Terms like *bright* and *dull* were commonly used as descriptors of children’s abilities and instructional needs during this period. Whipple’s study of bright children resulted in the term gifted, with corresponding educational recommendations some of which remain relevant today (e.g., curriculum compacting or telescoping) (Whipple 1919). There are no records to describe Whipple’s rationale for choosing the term gifted; however, a reasonable explanation is suggested by Whipple’s beliefs in hereditarianism and innate ability. As a gift is something innate to an individual, much like their eye color or height, so too was their intellect (Whipple 1919; Jolly 2007).

### 2.2. Developments in Gifted Education Policy, 1950s–1970s

#### 2.2.1. Sputnik and the National Defense Education Act

The launch of the Sputnik satellite in 1957 by Russia set off worries about the competitiveness (or lack thereof) of students in the United States. In response, the federal government focused heavily for several years on the development of academic talent among U.S. students, passing the National Defense Education Act in 1958 (NDEA; P.L. 85–684). This focus led to the development of Advanced Placement (A.P.) coursework; the successes of the space program, culminating in the Moon landing; and the continued expansion of higher education that had begun with the G.I. bill following the end of WWII. The work around NDEA also shifted conversations about education to a focus on academically talented youth and suggested that a greater number of students should be identified to support the U.S.’s role in the Cold War (Tannenbaum 1983).

#### 2.2.2. The Marland Report

The congressionally mandated Marland Report (1972) was the first federally sponsored report to address gifted students. It came at a time of increasing federal spending and attention to remedying social issues, as well as being during the latter years of the war in Vietnam and the progress and setbacks of the Civil Rights movement. By the late 1960s and early 1970s, both gifted and special education benefitted from this movement’s attention to goals of equality under the law and in practice (Jolly 2018; Jolly and Matthews 2014). Though mostly remembered today for having proposed the first federal definition of giftedness, it is notable that Marland also wrote in the report’s executive summary that “disturbingly, research has confirmed that many talented children perform far below their intellectual potential … this loss is particularly evident in the minority groups who have in both social and educational environments every configuration calculated to stifle potential talent” (p. 6). Marland further suggested that a minimum of three to five percent of the school population should be considered gifted and talented, but that “existing services to the gifted and talented do not reach large and significant subpopulations (e.g., minorities and disadvantaged) and serve only a very small percentage of the gifted and talented population generally” (p. 10).

In hindsight, in addition to the importance of the first federal definition of giftedness, Marland was right to emphasize schools’ failure in serving gifted students in general, and particularly the failure to educate students with gifts and talents from “minority groups” (p. 6). In addition to incorporating creativity, Marland’s definition expanded the idea of giftedness to include leadership and to allow for the domain specificity that we now know is characteristic of many students identified as gifted (Bernstein et al. 2019). Psychomotor giftedness was removed in 1978, as it was determined that enough resources were being provided to athletic talent (Jolly 2009). More importantly, just as the term ‘gifted’ is no longer nuanced enough for current purposes, Marland’s lumping of students into the catch-all of ‘minority’ did not provide enough detail to guide specific reforms at that time. Today, these students likely would be described with more focused and potentially overlapping categories such as students with dual or multiple exceptionalities; students from low-income or low-socioeconomic homes; students from specific linguistic and/or cultural or geographic backgrounds; students whose families are refugees or are experiencing homelessness; and the like. Given that he was writing during the height of the Civil Rights movement and near the tail end of the federal government’s War on Poverty as part of Lydon B. Johnson’s Great Society, one might have expected more emphasis devoted to issues of economic and educational parity.

### 2.3. Desegregation, State Policies, and Giftedness

The Brown vs. Board of Education decision in 1956 desegregated public schools, yet many districts throughout the United States did not actually desegregate their schools until the early 1970s. Prominent efforts to resist desegregation while satisfying the letter of the law included the development of separate city and county school districts drawn along racial lines and the establishment of secular and religious private schools to serve students whose parents had the financial means and desire to avoid sending their children to desegregated public schools. Magnet schools were originally proposed as a voluntary integration strategy, but while they have been largely successful on paper at achieving this goal, they also have created within-school segregation where it may not have existed previously. Other similar alternatives, particularly charter schools, have also contributed to the resegregation of schools in recent decades (Clotfelter et al. 2018).

Some authors critical of public education have also identified gifted programs as a way for schools to continue providing the same separate-but-equal segregated schools that were struck down in the Brown ruling. While a likely outcome in some school districts, relatively little direct evidence has been provided to support these assertions. Facts that suggest this was not a primary goal include the small amount of time that students identified as gifted are typically grouped with their intellectual peers—often less than 30 min per week—and the fact that gifted education legislation in many states was not codified until decades after desegregation had been accomplished.

Magnet schools were introduced as a desegregation tool and as a form of voluntary desegregation (George and Darling-Hammond 2021) but have found mixed success. Magnet schools remain a policy strategy in high-poverty and/or majority-minority school buildings located in core urban areas. In these cases, the typically high proportion of White students identified as gifted is apparently used as a tool to maintain school integration, raise average test scores in specific schools, and prevent the flight of affluent and White students and their families from urban schools to outlying, higher achieving suburban schools and districts.

There is evidence to suggest that the early use of gifted and talented programs to reduce White flight had a limited impact. Neighborhood transitions between 1970 and 1980, as detailed by demographers Denton and Massey (Denton and Massey 1991; Massey and Denton 1998), indicated White families left areas as multiple minorities groups gained a presence. As the utilization of this school provision by White families was limited, these gifted programs were often underfunded and resourced. Marland highlighted the need to identify and serve a greater number of students from underserved backgrounds, but this discussion was derailed by school districts’ use of gifted and talented programs to prevent White flight. Instead of the focus being on gifted and talented students from underserved backgrounds, the focus turned to keeping White students enrolled. Anecdotally, Dweck (2000) hypothesized that the gifted label conveys prestige or sense of being special; the vaguely defined meaning and simultaneous sense of entitlement the label offers their child are apparently also quite desirable to some parents. Needless to say, these are appalling reasons even if they do help explain the gifted label’s persistence.

The shifts in school population during and in response to desegregation and the systemic social issues that remain are beyond the scope of this paper; however, these issues exemplify a certain precariousness which often plagues gifted education services.

### 2.4. Developments in Gifted Education Policy, 1980s–1990s

#### 2.4.1. The Reagan Era, Block Grants, and the Nation at Risk Report

Federal funding for gifted education provided in P.L. 95-561, the Gifted and Talented Children’s Education Act, totaled USD 18.3 million between 1978 and 1981 following the gains realized following the advocacy and attention garnered by the Marland Report. The Act allowed state departments of education to apply for grant funding designed to improve gifted education through research, professional learning, planning, and programming (Jolly and Robins 2016). The Carter administration had elevated the Office of Education to a Cabinet position in 1979, providing a seat at the table for all education needs at highest level of government in the United States (Jolly and Robins 2016).

In an almost whiplash reaction, with Ronald Reagan’s election as President in 1980, his administration led a concerted effort to shrink the footprint of the federal government in American education (Verstegen 1990; Verstegen and Clark 1988). Congress opposed the elimination of the Cabinet position, but the Office of Gifted Education did not survive the severe budget cuts. Forty-three programs were rolled into one mega block grant for elementary and secondary education via the Education Consolidation and Improvement Act. Block grants to states were packaged as a means of returning autonomy to states in exchange for less accountability to the federal government. Less radical in its approach to education spending, Congress authorized more money than requested by the President’s office but still well under what had been allocated during the Johnson and Carter administrations (Verstegen 1990).

Gifted education received no federal funding from 1981 to 1988, as federal special program funding was reduced 76% from 1980 levels (Verstegen and Anthony 1988; Jolly and Robins 2016). Educational research (70%), Impact Aid programs (63%) (e.g., free and reduced lunch), compensatory education (25%), and bilingual education (54%) also incurred significant reductions during this same period (Verstegen 1990). These budget cuts deepened systemic social issues that had only begun to be addressed during the Johnson Administration’s Great Society legislation (Jolly 2018). Special education programs during this time were reduced by just 6% thanks to a concerted effort led by large advocacy and parent groups (Kauffman 1989).

In 1983, *A Nation at Risk* (National Commission on Excellence in Education 1983), produced by an 18-member panel impaneled by Secretary of Education Bell, sought to expose the educational failings of the United States. Scholars in gifted and special education who were invited to Harvard University to testify as part of the Commission’s work included Alexinia Baldwin, John Feldhusen, James Gallagher, Joseph Renzulli, Julian Stanley, and Abraham Tannenbaum (Jolly 2018). The report’s eight-point plan underscored the need to improve the quality of American schools and support of excellence. The recommendations of the report successively highlighted the needs of gifted and talented students. A compelling indicator of risk for gifted students noted “over ½ of the population of gifted students do match their tested ability with comparable achievement in school (National Commission on Excellence in Education 1983, para. 10). The field of gifted education routinely identifies *A Nation At Risk* as an important moment in its history. While gifted education advocates used report recommendations to gain access to services and resources, overall whether any systemic gains resulted is difficult to evaluate (Jolly 2018).

*A Nation At Risk* changed the messaging of Reagan’s reelection campaign but it did not result in any meaningful changes to federal education funding. The end of Reagan’s fiscal policies and renewed interest in gifted education federal funding could not replace the types of activities available under P.L. 95-561.

#### 2.4.2. The National Excellence Report (1993)

After several punishing years during the Reagan administration, gifted education turned a corner beginning in 1987 with the Congressional ratification of the Jacob K. Javits Gifted and Talented Children and Youth Act (P.L. 100-297 or the Javits Act), which provided $7.9 million USD from FY 1988 to 1993 with a focus on historically underserved students in gifted and talented programs. The Javits priorities shifted the focus from those offered in P.L. 95-561. The Javits program’s focus on research and effective dissemination has varied over time. The Act included the establishment of the National Research Center on the Gifted and Talented, yet research funding beyond the Center is almost non-existent in its most recent iteration, whose focus included research but also supported programs serving students and technical assistance and dissemination efforts (see https://oese.ed.gov/offices/office-of-discretionary-grants-support-services/well-rounded-education-programs/jacob-k-javits-gifted-and-talented-students-education-program/; accessed on 3 October 2022).

The *National Excellence Report: A Case for Developing America’s Talent* (Ross 1993), published only a decade after *A Nation At Risk*, once again focused attention toward the educational issues faced by gifted students. Initiated by the Office of Educational Research and Improvement and supported by a steering committee that included practitioners and scholars from the field of gifted education (James Gallagher, Mary Frasier, Sally Reis), the report highlighted “a quiet crisis” for gifted education in America’s schools in the absence of any special interventions to meet the cognitive needs of gifted students. The report also brought renewed attention to the historical problem of the lack of diversity in gifted education programming. Vestiges from *A Nation At-Risk*’s eight-point plan could be recognized in the eight *National Excellence* recommendations to improve educational opportunities for gifted children, which included to (a) set challenging curriculum standards; (b) provide challenging opportunities to learn; (c) increase access to early childhood education; (d) increase opportunities to learn for disadvantaged and minority students; (e) broaden the definition of giftedness; (f) emphasize teacher development; and (g) increase the international educational standing of the United States. These recommendations and others were also provided as focal areas for future research by those in the field (Jolly and Kettler 2008). However, due in large part to the direction and monies provided by the Javits Act, in the 10 years immediately following the National Excellence report only one of its recommendations received any sustained attention—underserved minority students (Jolly and Kettler 2008).

### 2.5. Developments in Gifted Education Policy in the 2000s

#### 2.5.1. The Nation Deceived Report

School settings, intellect and abilities, and to a somewhat lesser extent research on curriculum and policy were the main foci of work between the *National Excellence* and the next report of note in 2004 (Jolly and Kettler 2008). The early 2000s also saw an increased emphasis on testing with the passage of the No Child Left Behind Act (107th U.S. Congress 2001), but the law’s narrow focus on achieving minimum proficiency standards led many teachers to ignore the needs of their high-ability students (Thomas B. Fordham Institute 2008).

The next nationally disseminated report to be related closely to gifted education was the *Nation Deceived* report of 2004 (Colangelo et al. 2004). Unlike previous reports, this one did not originate from the federal government and was produced to correct popular misconceptions about the effectiveness of acceleration as an intervention for advanced learners. The report was notable for its well-organized and far-reaching public dissemination, but evidence in the follow-up report (2015) suggested that as with the other national reports, there were few changes in the schools despite the strong empirical evidence the report had provided in support of academic acceleration as an intervention for gifted and high-achieving children.

The *Nation Deceived* report and its follow up (Assouline et al. 2015) offer a sobering example of a key problem facing gifted education as a field, which is the distinct disinclination of schools to adopt research-supported best practices for their students identified as gifted. Despite the existence of ample evidence in favor of academic acceleration in the 2004 report and even more recently (Steenbergen-Hu et al. 2016), the practice is widely ignored by schools. Ability grouping, which is perhaps the next best-supported practice in gifted education, is likewise implemented relatively rarely. This failure of schools to use practices that are known to be effective is not the case in other areas of special education, where the use of evidence- or research-based best practices is often mandated in policy (see Matthews and Hujar 2020 for discussion of different levels of evidence in education research).

#### 2.5.2. The Every Student Succeeds Act

The passage of the Every Student Succeeds Act (ESSA) in 2015 authorized for the first time the use of Title I and Title II funds to support the needs of gifted students. The law permitted Title I funds to be spent on the identification of gifted students and Title II professional development monies to be expended to improve the competencies of teachers working with gifted students. Although considered an improvement, the choice of how these monies are spent remained up to the school administrator or district’s discretion. Thus just as was the case for block grants at the state level, in the absence of a mandate, relatively few districts appear to have used these funds to support the education of their students with gifts and talents.

We have mentioned these specific reports and related developments since *Marland* because of their prominence within the gifted education field and its literature. Even though these more recent reports do not directly use the word gifted in their titles, they have been influential in the field’s historical development. However, there also have been developments within the field that have not received as much attention from audiences outside of it. One salient internal development has been the discussion of talent development versus giftedness.

#### 2.5.3. Talent Development vs. Giftedness

The NDEA and the Great Talent Hunt initially focused discussion on the potential of students and how students’ raw talent could be used as a weapon in the Cold War (Tannenbaum 1983). Later, talent development was suggested as a potential descriptor to replace gifted education (Feldhusen 1995). Reframing ‘gifts’ as ‘talents’ underscores both the domain-specific nature of these abilities and that their extent should be viewed as dynamic rather than static or preordained. This proposed renaming was not well received by the field when it was initially proposed, but it seems to have encountered less resistance when it later was reframed with a more solid basis in psychological science by Subotnik and her colleagues (Subotnik et al. 2011; see further discussion in Section 2.8 below).

### 2.6. Differing Evidence of Effectiveness

Do gifted education services provide successful interventions, in the way that special education or programs for students learning English do? Students exiting special education or other compensatory programs are viewed as successful. While Marland stated that the evidence showed gifted and talented programming to be effective, some more recent studies have failed to confirm this (e.g., Adelson et al. 2012). Other studies with more nuanced data have shown that gifted programming does work, at least for some students (Card and Giuliano 2014). Thus, the differences observed in effectiveness may be due to fragmentary programming and incomplete sources of data rather than providing strong evidence that gifted services are ineffective; but regardless, these varied findings on effectiveness and the lack of consensus in the field about what the outcomes of successful gifted programming should be are both barriers to effective advocacy on behalf of students with gifts and talents.

### 2.7. Special Education, Gifted Education, and Labels

Special education includes 13 categories of disabilities that are defined under U.S. federal law. Kanaya (2019) provided a succinct summary:

At the individual level, a student’s performance on intelligence measures is used in many decisions that affect his or her day-to-day experience in the classroom. Much of this is due to the Individuals with Disabilities Act (IDEA; initially the Education for All Handicapped Children Act), which is more commonly referred to as special education [2]. Under IDEA, all students who meet the criteria for the 13 categories in the Code for Federal Regulations (CFR) [3] are entitled to ‘free and appropriate’ educational services. Each diagnostic criteria, however, requires an IQ test. Furthermore, every student who receives services is required to undergo a re-evaluation at least every three years, thereby making school children in special education one of the most heavily tested and re-tested populations in the United States on IQ tests.

Giftedness is not one of these categories; the various federal definitions of giftedness beginning with Marland have been suggestions rather enacted law or policy at the federal level. However, the broader term exceptionality typically includes both special and gifted education under its umbrella. Thus, the largest national advocacy organization for students with exceptionalities is the Council for Exceptional Children; among its 18 special interest-focused Divisions is CEC-TAG, The Association for the Gifted (both authors of this manuscript currently hold leadership roles in CEC-TAG). Other advocacy organizations that primarily support gifted learners, such as the National Association for Gifted Children (NAGC), generally only focus on special education in the context of students identified as having dual or multiple exceptionalities, typically giftedness and one or more of those special education diagnoses that can co-exist with high academic ability. Giftedness typically is defined at the state level (Rinn et al. 2020), and state policies and support for learners labeled gifted consequently vary quite a bit more than the federally defined labels and categories used in special education do.

Labels serve a useful function in that they help guide the allocation of funding in schools and other organizations, ensuring that monies address specific, recognized needs and populations. However, compensatory education labels also have prescriptive utility. Special education services may be based on specific diagnoses such as dyslexia, processing disorder, or the like. In contrast, simply labeling a student with the broad term ‘gifted’ does not effectively inform service delivery at a fine-grained level. IQ testing provides a legitimate basis for an initial diagnosis in both gifted and special education, and it is a strong (though far from infallible) predictor of subsequent achievement (Deary et al. 2004, 2008; Józsa et al. 2022; Wai et al. 2018), but its results only should be used to include students in gifted education programming—not to exclude them from it, as is all too frequently the case. In addition to the gifted label not reflecting advances in intelligence research, the label is also ineffective in informing school programming and student services as the label is nearly meaningless given its varied and inconsistent interpretations.

Labels also can be stigmatizing. Perhaps counter-intuitively, this also holds true for the gifted label, which might otherwise be thought to carry positive associations. In a study by Matthews and colleagues (Matthews et al. 2014), a majority of parents of children identified as gifted reported that they avoided use of the word gifted because they feared its use would lead others to judge their children in a negative manner.

Over time, the field of special education has adapted to changing societal mores by updating its preferred terminology—for example ‘mental retardation’ is no longer used (Nash et al. 2012). Interestingly, this particular change originated in self-advocacy efforts by members of the population with this diagnosis, a position that subsequently was adopted by scholars and by government and advocacy organizations. Other terms like ‘moron’ and ‘imbecile’ that once were widely used and had specific meanings in the early days of intelligence testing have likewise been abandoned over time, in favor of less-pejorative and person-first language. To date, students identified as gifted have yet to engage in such self-advocacy despite their willingness to express their reservations about the term (Hertzog 2003).

### 2.8. Research in Other Related Fields

Given the lack of empirical evidence addressing the effectiveness of gifted programming, one might wonder, why have researchers in gifted education not devoted more effort to collaborating with scholars working in other fields? There have been a few examples of how research conducted outside the field of gifted education has helped inform understanding within the field (Makel et al. 2020) and it is instructive to consider these efforts.

One well-publicized example of this kind of interdisciplinary work is provided by Subotnik et al.’s monograph, “Rethinking Giftedness and Gifted Education: A Proposed Direction Forward Based on Psychological Science” (Subotnik et al. 2011) in which the authors synthesized findings about giftedness to make the case for a domain-specific perspective and a focus on talent development as constituting the proper scope of gifted education efforts. A domain-specific focus is consistent with the pattern of results obtained from IQ tests and other related measures (e.g., the SAT; see Lubinski 2016; Lubinski and Benbow 2021). They further asserted that “outstanding achievement or eminence ought to be the chief goal of gifted education” (p. 3). From the perspective of furthering the dialogue in gifted education, these authors’ synthesis of findings from psychology with those from education offered a productive example of how this might be accomplished.

In a second example, research by economists has further informed the understanding of disproportionality in gifted identification and services and has brought a new perspective on these issues to scholars in the field (Peters and Matthews 2016). Some subsequent research has included direct collaborations between scholars in gifted education and economics. However, the focus and goals of economists often differ from those of educators, suggesting that caution is warranted in seeking to more closely connect gifted education to research in disciplines of study that are more distal to education.

The advances produced so far through cross-disciplinary collaboration have been rewarding and instructive. They suggest that collaboration with other fields, for example neuroscience, could also yield different approaches to identification or more nuanced approaches to providing effective programming and services (Makel et al. 2020). This approach also has been influential in the development of special education, where (for example) medical science has informed understanding of some disabilities; but see (Cornett and Knackstedt 2020) for a critical perspective on such borrowing.

Lastly, despite a move toward greater recognition of non-cognitive factors and growing awareness of its limitations in identifying students from groups traditionally underserved in gifted programming, IQ remains widely used in many states’ definitions of giftedness (Rinn et al. 2020). There are a number of areas still being studied by intelligence researchers that have the potential to inform gifted identification practice and, potentially, to inform the search for a more suitable label for the students identified via IQ testing as needing more advanced learning opportunities to fulfill their potential. For example, a recent Special Issue of this journal focused on the executive processes that may underly *g*. Conway and colleagues (Conway et al. 2021) and others in the issue discussed process overlap theory, a sampling model of *g* that conceptualizes intercorrelations between tests as being due to an overlap in multiple domain-general and domain-specific cognitive processes. Such a model has the potential to inform individualized educational interventions for developing learners’ strengths, the primary focus of gifted education services, as well as to develop learners’ compensatory abilities in areas of under-performance, the focus in other areas of exceptional student education.

## 3. Discussion and Conclusions

The label issue for gifted education still fails to reflect what we have learned over a hundred years of scientific study. Over time our understanding of learning, teaching, personality, and the influence of non-cognitive factors on educational attainment has grown dramatically, but the identification practices and terminology of gifted education have fallen drastically behind the progress made on these same issues in the related field of special education.

In the best of cases, a label indicates needed services without conveying prejudice or other ancillary meanings. Labels used in special education have become more nuanced and prescriptive over time, whereas in gifted education there are fewer branches—a cactus rather than a tree—meaning that the general gifted label is far less useful for purposes of service delivery than a special education label (e.g., dyslexia or a specific learning disability in numerical processing). This is true even when the gifted label is further divided into identification categories based on academic achievement vs. intellectual potential, or even into reading or English language arts vs. mathematics. In other words, the utility of the gifted label remains on par with the utility of early special education terms like ‘moron’—which is to say, it still has little relevance to service delivery.

Marland’s report and the others that have followed were natural inflection points to update the gifted label, but this was not the intention of these reports. The first comprehensive report of its kind, the Marland report described then-current educational services for students identified as gifted, evaluated the extent of programming and services, and offered recommendations for new programming. Diversity and inclusion also featured as main issues to be addressed (Marland 1972; Jolly 2018). Though not the central focus of this paper, diversity and inclusion have remained central issues in the field to this day, as a recent Special Issue of the journal *Gifted Child Quarterly* confirmed (Worrell and Dixson 2022).

Following Marland, later reports continued to draw attention to the lack of educationally appropriate services provided to students with abilities and needs beyond those of the typical classroom learner. It was undeniably important at that time, as it still continues to be, to raise awareness of the unique learning requirements of students identified as gifted. However, this focus means these reports’ authors have not focused closely on the relationship between labels and services, or suggested a pathway forward for correcting the limitations associated with the term gifted. For example, can services rather than students be labeled, as suggested by Peters and his colleagues (Peters et al. 2014)? Should interventions for students identified as gifted be developed using a Response to Intervention (RtI) or Multi-Tiered Systems of Support (MTSS) model (Coleman and Johnsen 2013; Stephens 2020)? If any of these changes were made, how might they affect the allocation of the already insufficient levels of funding (Rinn et al. 2020) that some states currently provide in partial support of gifted education services?

Marland’s report more than 50 years ago set off a chain of regular (if not intentional) reporting on topics of excellence and equity. However, the missing link common to all of these publications has been the funding, mandated policy or law, and personnel to enact the reports’ recommendations into practice. Javits funding at its highest (FY 2022) was $14.5 million, while the corresponding federal special education funding was over $14 billion or nearly one thousand times higher. There also have been notable gap years in which the Javits program received no funding at all, or did not fund new proposals, which has never been the case in special education research. In special education the funding, policy mandate at the federal level, and research into evidence-based practices are all tied together in a system that has been extremely effective in securing appropriate services for students in special education but the failure to include giftedness with the other categories of exceptionality recognized under federal law has left gifted education mostly to its own devices for the past 60 years. Despite the map forward provided by Marland and subsequent reports, the field has been unable to realize its potential. This is most notably reflected in the ongoing use of the broad label of gifted. Special education organizations, practitioners, and families have not had the same difficulty in replacing archaic terms with newer ones as understanding of the impact of such labels has grown.

Misunderstanding persists regarding the utility of current IQ tests and what their scores can reveal about high intellectual ability, despite the several longitudinal studies that convincingly have demonstrated the predictive power of IQ for a variety of positive life outcomes. For example, Lewis Terman’s work in California and the Scottish Mental Survey both illustrated a positive correlation between IQ and longevity (Deary et al. 2008; Holahan 2021). Wai and Brown (Wai and Brown 2021) using a sample of 48,558 individuals from the US and the UK found that cognitive ability in youth correlated with several positive outcomes in educational, health, social, and occupational attainments. Moreover, the contributions of genetic influences to measured IQ scores appear to increase with age; this may suggest that school-based programming has more influence on younger learners, a view that would support the effectiveness of gifted education and other school-based interventions, or perhaps there are selection effects, motivational differences, or other extraneous variables that influence this relationship. Regardless, schools offer the primary source of the extensive practice that is needed to transform intellectual potential into actualized talent (Subotnik et al. 2011), yet the positive outcomes consistently associated with high cognitive ability are not typically considered by schools in making decisions about student placement, instructional grouping, or curriculum (Wai 2014; Wai et al. 2018).

The United States has had a particularly enigmatic relationship with intelligence testing and its applications in social and educational contexts. Leaders during the Progressive Era sought to promote organization and orderliness in every aspect of society. Aided by psychologists such as Lewis Terman, the IQ test provided a mechanism for the sorting of students and others—including military recruits during the first World War—by mental ability, and thereby a foundation on which the first gifted and talented programs were established (Chapman 1981; Fass 1980; Jolly 2018). IQ testing was also used in other, more insidious ways. Eugenic societies thrived during the first several decades of the 20th century as they pursued the goal of bettering human society through the control of reproduction, largely among those from non-White racial backgrounds, the physically or mentally ill, and those considered feebleminded, criminal, or socially deviant (Wilson 2002). For example, low IQ scores were used to identify individuals for forced sterilization campaigns carried out throughout the United States and continuing until as recently as the 1970s. This misguided and reprehensible use of IQ test results likely has fueled the ongoing narrative among some writers that claims IQ tests are intentionally designed to be racially biased (rather than the more reasoned view that their differential results reveal a lengthy history of systemic discrimination and related societal inequities). In gifted education these intertwined ideas likely have impeded a greater acceptance of recent research related to IQ and intelligence and thereby also have limited the field’s ability to consider labels that could have greater utility than the term gifted.

In 2022, a Special Issue of *Gifted Education International* was devoted to the “terminological controversies” in the field. One of the main takeaways across the Special Issue’s constituent manuscripts was that any effective term or label must provide clarity (Sternberg and Desmet 2022). We agree with this conclusion, but we would also insist that any such term must also imply a specific educational need. As we have demonstrated in the present review, scientific, political, and social forces have created an unpredictably predictable pattern of concern, contempt, and indifference that has left the field largely ineffectual in its quest to find more appropriate practices or even a label suitable for use in the 21st century. The field of special education has been much more effective at revising labels to reflect scientific findings, providing a greater balance between within-child and environmental factors, developing defensible goals for children with special needs, and reconciling debates among stakeholders regarding the specificity and appropriateness of the terms used to describe students and their educational needs (Farrell 2014). Any new term or terms to replace the word ‘gifted’ would need to have a more diagnostic or prescriptive meaning and be far more domain specific. Fear of what might happen in the absence of the word gifted also, at least anecdotally, seems to be a barrier to progress, and this also has led to suggestions to do away entirely with labeling students (e.g., Borland 2005). Labeling programs rather than students has also been suggested (Peters et al. 2014), but the label also serves to direct attention and funding to meeting specific learner needs (Kauffman 1989) and this suggests that some form of label may always be needed. As evidenced from the conversion of specific funding to block grants under the Reagan administration, services tend to vanish when they are combined into a larger general budget rather than being named specifically in policy. A more specific label or labels also would be helpful to the general education classroom teacher, who would then have a better understanding of what services or content and strategies might be effective for a given student.

In closing, we urge those who wish to support the needs of students currently or potentially labeled as gifted learners to continue thinking about how to improve the label in a manner that would make more evident these learners’ specific individual and collective differences and needs from typical learners, while also being more palatable to policy makers and the public at large than the term gifted currently is.

## Data Availability

Not applicable.
